# Perceived weight discrimination and chronic biochemical stress: A population‐based study using cortisol in scalp hair

**DOI:** 10.1002/oby.21657

**Published:** 2016-10-14

**Authors:** Sarah E. Jackson, Clemens Kirschbaum, Andrew Steptoe

**Affiliations:** ^1^ Department of Epidemiology and Public HealthUniversity College LondonLondonUK; ^2^ Department of PsychologyTechnische Universität DresdenDresdenGermany

## Abstract

**Objective:**

There is increasing evidence for weight‐based discrimination against persons with obesity. This study aimed to examine the physiological impact of perceived weight discrimination on cortisol in hair, an indicator of chronic stress exposure.

**Methods:**

Data were from 563 nonsmoking individuals with obesity (body mass index, BMI ≥30 kg/m^2^) participating in the English Longitudinal Study of Ageing. Experiences of discrimination were reported via questionnaire, and hair cortisol concentrations were determined from the scalp‐nearest 2‐cm hair segment. Height and weight were objectively measured. ANCOVAs tested associations between perceived weight discrimination and hair cortisol concentration overall and by degree of obesity. All analyses were adjusted for age, sex, ethnicity, socioeconomic status, and BMI.

**Results:**

Mean hair cortisol concentrations were 33% higher in those who had experienced weight discrimination than those who had not (mean log pg/mg 1.241 vs. 0.933, *F* = 12.01, *P =* 0.001). The association between weight discrimination and hair cortisol was particularly pronounced in individuals with severe (class II/III) obesity (1.402 vs. 0.972, *F* = 11.58, *P =* 0.001).

**Conclusions:**

Weight discrimination is associated with the experience of stress at a biological level. Chronic exposure to elevated levels of cortisol may play a role in generating a vicious circle of weight gain and discrimination and contribute to obesity‐associated health conditions.

## Introduction

The rising prevalence of obesity over recent decades has been paralleled by growing evidence of weight‐based stigma and discrimination 1. Negative stereotypes of individuals with obesity as lazy, weak willed, and personally to blame for their weight are pervasive across multiple settings including employment, healthcare, education, media, and interpersonal relationships 2. In national surveys in the UK and US, up to 14% of individuals with moderate obesity (body mass index, BMI 30‐34.9 kg/m^2^) and 43% with severe obesity (BMI ≥35 kg/m^2^) reported being treated unfairly because of their weight 1, 3.

The experience of weight discrimination may be interpreted by the body as a social stressor, causing activation of the hypothalamic‐pituitary‐adrenal (HPA) axis and resultant release of the hormone cortisol. Weight discrimination is associated with a host of adverse psychological consequences, including depression and anxiety disorders 4, low self‐esteem 5, and poor quality of life 6, which are also known to be related to stress (e.g., Ref. 7). Many obesity‐associated comorbidities are stress‐related and may be attributable, at least in part, to the stress of discrimination 8. The experience of stress and consequent rise in cortisol levels are associated with hypertension, dyslipidemia, insulin resistance, suppression of the immune system, and other disturbances to metabolic and endocrine functions 9. Weight discrimination has also been shown to be related to increased blood pressure 10, chronic inflammation 11, poorer self‐rated health 12, greater disease burden 12, and a decline in physical health over time 12.

Importantly, in addition to having emotional and disease‐related consequences, an association between weight discrimination and chronic stress may be implicated in the perpetuation of obesity. Previous studies have shown that people who perceive weight discrimination tend to gain weight over time 13 and are more likely to continue having obesity 14. Chronic stress arising from experiences of discrimination may promote the accumulation and retention of excess weight via effects of cortisol on fat deposition and food intake. Chronic exposure to physical and psychological stressors increases deposition of visceral fat 15. Cortisol activates glucocorticoid receptors, which are densely concentrated in visceral fat depots. When insulin is present, cortisol promotes storage of triglycerides in visceral adipose tissue resulting in increased abdominal fat 15. Studies on stress and weight discrimination have both shown evidence of upregulation of appetite and increased cravings for “comfort foods.” People report eating more when they are stressed 16, and studies in patient and community samples have shown associations between weight discrimination and overeating 17, 18. In experimental studies, infusion of glucocorticoids and artificially induced stress have been shown to increase *ad libitum* calorie intake 19, as has exposure to stigmatizing messages about obesity 20, 21. Weight‐stigmatizing stimuli increase the stress response and reduce executive control 10, which may inhibit regulation of food intake. Functional MRI studies have shown reduced sensitivity of central food reward circuits in response to stress, which may increase the drive to consume comfort foods 22. In times of stress, people tend to consume more palatable, energy‐dense foods even if their total energy intake is unchanged 23. A recent study showed that weight discrimination was associated with greater consumption of convenience food, such as fast food, snack food, and sugar‐sweetened beverages, but was unrelated to intake of healthy food 24.

In light of these observations, two recent theories have posited that weight stigma is a stressor that stimulates cortisol secretion and thus promotes weight gain and abdominal adiposity and inhibits weight loss. Tomiyama's cyclic obesity/weight‐based stigma model proposes a positive feedback loop whereby weight stigma produces stress and accompanying physiological, emotional, and behavioral responses, which in turn lead to weight gain, subsequently increasing vulnerability to weight stigma 25. Hunger et al.'s social identity threat model suggests that weight‐based social identity threat increases physiological stress, inhibits self‐regulation, reduces psychological well‐being, and leads to avoidance of stigmatizing situations and engagement in unhealthy weight loss behaviors in an effort to escape the stigma 26.

Two experimental studies have shown changes in salivary cortisol in response to weight stigma. Schvey et al. 27 randomized participants to view either a weight‐stigmatizing or neutral video and measured salivary cortisol before and after viewing; significantly greater cortisol reactivity was observed in those in the stigmatizing condition. Himmelstein et al. 28 found an association between weight stigma and cortisol reactivity using an active weight‐stigmatizing manipulation, but only in participants who perceived themselves as heavy. A small observational study of patients with overweight and obesity seeking treatment for stress eating also provides evidence of an association between weight discrimination and stress, with scores on a questionnaire inventory of experiences and consciousness of weight stigma positively related to cortisol levels in saliva and serum 29.

While this research provides preliminary evidence for immediate physiological stress responses to weight stigma, there have been no published studies examining associations between weight discrimination and chronic stress. This is important given evidence that acute stress serves as a protective, adaptive response whereas chronic stress can have a damaging effect on the body 30. Assays of cortisol in saliva and blood can be accurately used to measure momentary cortisol concentrations at the time of sampling, but fluctuations in cortisol levels due to the circadian rhythm, pulsatile secretion, and daily variation arising from situational factors (e.g., diet, environmental stressors, infection) mean they are not well suited for capturing chronic cortisol concentrations. A novel method of measuring cortisol in scalp hair has gained popularity over the past decade as it provides an indication of mean exposure to free cortisol over 1 month per 1 cm of hair 31. Considerable evidence now supports the validity 32 and test–retest reliability 33 of hair cortisol concentration as a marker of chronic cortisol exposure.

This study aimed to examine the association between weight discrimination and chronic cortisol exposure using scalp hair in a population‐based sample of people with obesity. We hypothesized that perceptions of weight discrimination would be associated with chronic stress, evidenced by elevated hair cortisol concentration.

## Methods

### Study population

Data were from the English Longitudinal Study of Ageing (ELSA), a longitudinal panel study of men and women aged 50 years and older living in England. The original sample was recruited from an annual cross‐sectional survey of households in 1998, 1999, and 2001 and has been periodically refreshed to ensure the full age range is maintained. Comparisons of sociodemographic characteristics with the national census indicate that the sample is broadly representative of the English population 34. Full details of the methods of data collection are available at www.elsa-project.ac.uk. The first wave of data were collected in 2002/2003 and participants have since been followed up every 2 years. At each assessment, participants complete an interview and questionnaires, and in alternate (even) waves nurses visit participants in their homes to obtain objective measurements of health status, including height and weight. To date, Wave 5 (2010/2011) is the only assessment that has included questions on discrimination and the Wave 6 (2012/2013) nurse visit is the only wave in which hair samples were collected for the assessment of cortisol. For our analyses, we used these data plus demographic and anthropometric data collected in Wave 6. Complete data were available for 2,001 participants. We restricted our sample to nonsmoking participants with obesity (BMI ≥30 kg/m^2^), resulting in a sample for analysis of 563 men and women. We excluded smokers because smoking interferes with cortisol levels, and we chose not to include participants with a BMI in the overweight range (BMI 25‐29.9 kg/m^2^) because previous analyses in this cohort have shown very low prevalence (<1%) of perceived weight discrimination in this group 3. Participants gave full informed consent and ethical approval was obtained from the National Research Ethics Service.

### Assessment of weight discrimination

Questions on perceived discrimination were based on items developed and used widely in other longitudinal studies, notably MIDUS and the Health and Retirement Study 1, 14. Participants were asked how often they encounter five discriminatory situations: “*In your day‐to‐day life, how often have any of the following things happened to you*: 1 *you are treated with less respect or courtesy;* 2 *you receive poorer service than other people in restaurants and stores;* 3 *people act as if they think you are not clever;* 4 *you are threatened or harassed; and* 5 *you receive poorer service or treatment than other people from doctors or hospitals*.” No specific time frame for experiences of discrimination was indicated. Response options were on a 5‐point scale ranging from “never” to “almost every day.” Because data were highly skewed, with most participants reporting never experiencing discrimination, we dichotomized responses to indicate whether or not respondents had ever experienced discrimination in any domain (never vs. all other options) 3. A follow‐up question asked participants who reported discrimination in any of the situations to indicate the reason(s) they attributed to their experience from a list of options including weight, age, gender, race, physical disability, and sexual orientation. Participants could attribute more than one reason to their experiences of discrimination. Those who attributed any experience of discrimination to their weight are treated in our study as cases of perceived weight discrimination. This measure has been used successfully to track trends in weight discrimination over time 1, and we have previously used it to evaluate the prevalence of weight discrimination and associations with weight change and psychological well‐being in this cohort 3, 6, 13.

### Hair sample collection and analysis

A lock of hair measuring at least 2 cm in length and weighing at least 10 mg was collected from the posterior vertex of all consenting participants, cut as close to the scalp as possible. Participants were ineligible for hair sampling if they were pregnant, breastfeeding, had certain scalp conditions, were unable to sit with head remaining still, or had less than 2 cm of hair length in the posterior vertex scalp area. Full details of the hair sampling process are provided at http://www.elsa-project.ac.uk/uploads/elsa/docs_w6/project_instructions_nurse.pdf. The wash procedure and steroid extraction were undertaken using high‐performance liquid chromatography–mass spectrometry (LC/MS), as described by Gao et al. 35. Based on an average monthly hair growth of approximately 1 cm 31, the scalp‐nearest hair segment of 2 cm represents average cortisol accumulated over an approximate time span of 2 months before sampling. Information on hair‐specific factors that could affect hair cortisol concentration (dyeing or chemical treatment, such as perming or chemical straightening) was reported by participants.

### Measures of anthropometry

Weight was measured by nurses to the nearest 0.1 kg using portable electronic scales, and height was measured to the nearest millimeter using a portable stadiometer. Nurses recorded any factors that might have compromised the reliability of the measurements (e.g., participant was stooped/unwilling to remove shoes), and these cases were excluded. BMI was calculated from height and weight. Obesity was defined as class I (BMI 30‐34.9 kg/m^2^), class II (35‐39.9 kg/m^2^), and class III (≥40 kg/m^2^).

### Other variables

Age, sex, ethnicity, and socioeconomic status (SES) were included as control variables. We used household non‐pension wealth as an indicator of SES, because it has been identified as particularly relevant to health outcomes in this age group 36. Wealth was categorized into quintiles across all ELSA participants who took part in Wave 6. Smoking status was assessed with the question “*Do you smoke cigarettes at all nowadays*” (yes/no); current smoking was an exclusion criterion for the current study.

### Statistical analysis

Analyses were performed using IBM SPSS Statistics 23. We used weights to correct for sampling probabilities and for differential nonresponse and to calibrate back to the 2011 national census population distributions for age and sex. The weights accounted for the differential probability of being included in Wave 6 of ELSA and for nonparticipation in the nurse visit. Details can be found at http://doc.ukdataservice.ac.uk/doc/5050/mrdoc/pdf/5050_elsa_w6_technical_report_v1.pdf.

Hair cortisol data were log transformed to correct skewness. For descriptive purposes, Table 1 provides information on means and standard deviations (SD) in original units (pg/mg). Associations between descriptive characteristics and perceived weight discrimination were analyzed using *t*‐tests (continuous variables) and *χ* ^2^ tests (categorical variables). One‐way analysis of covariance was used to test for differences in mean cortisol levels between those who did and did not report weight discrimination. We repeated this analysis stratified by obesity class to test whether the association varied by degree of obesity; individuals with class II and class III obesity were combined for this analysis due to low numbers. We performed all analyses separately by sex to explore any sex differences, but as there were no notable differences in results, we only present the whole‐sample analyses here. Given statistical limitations inherent to the use of dichotomized variables, we tested associations with hair cortisol using continuous weight discrimination data as a sensitivity check. We also tested the association between hair cortisol and perceived discrimination in general (i.e., discrimination in any domain attributed to any characteristic, e.g., age, sex, race) using an aggregate variable of all forms of discrimination in order to explore whether observed associations were specific to weight‐related discrimination or applied to any type of perceived discrimination. All analyses were adjusted for confounding by age, sex, ethnicity, SES (indicated by wealth quintile), hair treatment, BMI, and the amount of time elapsed between Wave 5 and Wave 6.

**Table 1:** Sample descriptive characteristics—mean (SD) or % (*n*)

	Whole sample (*n* = 563)	Weight discrimination (*n* = 68)	No weight discrimination (*n* = 495)	*P*
**Age, years (*n* = 563)**	67.69 (7.31)	64.40 (6.43)	68.14 (7.32)	<0.001
**Sex (*n* = 563)**				
**Male**	37.1 (209)	9.6 20	90.4 (189)	0.160
**Female**	62.9 (354)	13.6 (48)	86.4 (306)	‐
**Ethnicity (*n* = 563)**				
**White**	98.4 (554)	12.1 (67)	87.9 (487)	0.928
**Non‐white**	1.6 9	11.1 1	88.9 8	‐
**Wealth quintile (*n* = 556)**				
**1 (poorest)**	15.1 (84)	17.9 15	82.1 (69)	0.067
**2**	20.1 (112)	16.1 18	83.9 (94)	‐
**3**	25.7 (143)	8.4 12	91.6 (131)	‐
**4**	21.4 (119)	7.6 9	92.4 (110)	‐
**5 (richest)**	17.6 (98)	14.3 14	85.7 (84)	‐
**BMI, kg/m^2^ (*n* = 563)**	34.11 (3.84)	37.21 (5.27)	33.69 (3.40)	<0.001
**Obesity class (*n* = 563)**				
**Class I**	69.3 (390)	6.9 27	93.1 (363)	<0.001
**Class II**	22.6 (127)	19.7 25	80.3 (102)	‐
**Class III**	8.2 (46)	34.8 16	65.2 30	‐
**Hair treatment (*n* = 562)**				
**Yes**	35.4 (199)	14.6 29	85.4 (170)	0.183
**No**	64.6 (363)	10.7 39	89.3 (324)	‐
**Hair cortisol, pg/mg (*n* = 563)**	35.36 (87.04)	75.19 (140.46)	29.88 (75.46)	0.011
**Elapsed time, years (*n =* 563)**	1.91 (0.13)	1.90 (0.17)	1.91 (0.12)	0.842

Unweighted data from Wave 6 of the English Longitudinal Study of Ageing. Numbers may not add up to the total sample number due to missing data. Valid percentages shown. Elapsed time is the time between data collection points (Wave 5 and Wave 6).

## Results

Characteristics of the study sample are summarized in Table 1. The sample comprised 563 nonsmoking individuals with obesity. Respondents were aged 67.7 years on average, 63% were female, and the majority (98%) were white. The mean BMI was 34.1 kg/m^2^, with 69% living with moderate (class I) obesity and 31% living with severe (class II/III) obesity. The mean hair cortisol concentration was 35.4 pg/mg (range 0.45‐549.60 pg/mg).

Perceived weight discrimination was reported by 12.1% of participants and increased with the degree of obesity, from 6.9% in those with class I obesity to 19.7% and 34.8% in those with class II and class III obesity, respectively (*P* < 0.001). Younger individuals were more likely to report experiencing weight discrimination (*P* < 0.001). Reports of weight discrimination did not differ significantly by sex (*P* = 0.160), ethnicity (*P* = 0.928), SES (*P* = 0.067), or hair treatment (*P* = 0.183).

Table 2 presents the results of one‐way analyses of covariance testing for differences in mean hair cortisol concentration by perceived weight discrimination. Overall, there was a significant association between perceived weight discrimination and hair cortisol concentration (*F* = 12.01, *P* = 0.001) after adjustment for age, sex, SES, ethnicity, hair treatment, BMI, and time elapsed between data collection points. Hair cortisol levels were on average 33% higher in individuals who reported perceived weight discrimination (adjusted mean 1.241 log pg/mg, 95% confidence interval [CI] 1.079‐1.404) than those who did not report weight discrimination (adjusted mean 0.933 log pg/mg, 95% CI 0.876‐0.991).

**Table 2:** Association between mean hair cortisol concentration and perceived weight discrimination overall and by degree of obesity—ANCOVA results

	Whole sample (*n* = 563)		Individuals with moderate obesity (*n* = 390)		Individuals with severe obesity (*n* = 173)	
	*F*	*η* ^2^	*F*	*η* ^2^	*F*	*η* ^2^
**Perceived weight discrimination**	12.01*	0.022	1.40	0.004	11.58*	0.070
**Age**	0.04	<0.001	1.31	0.004	1.58	0.010
**Sex**	0.10	<0.001	1.66	0.005	1.12	0.007
**Ethnicity**	1.28	0.002	1.47	0.004	0.00	<0.001
**SES**	0.05	<0.001	0.16	<0.001	0.22	0.001
**BMI**	2.00	0.004	0.01	<0.001	0.18	0.001
**Hair treatment**	0.02	<0.001	0.95	0.003	0.26	0.002
**Elapsed time**	0.80	0.002	0.66	0.002	0.19	0.001

*P < 0.01. All analyses were weighted for sampling probabilities and differential nonresponse.ANCOVA: analysis of covariance; SES: socioeconomic status. Elapsed time is the time between data collection points (Wave 5 and Wave 6).

**Figure 1:**
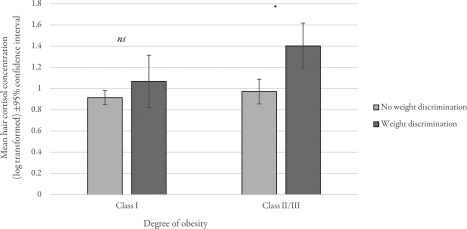
Mean hair cortisol concentrations by perceived weight discrimination in individuals with moderate (class I) and severe (class II/III) obesity. ns, nonsignificant; *P < 0.01. Data weighted for sampling probabilities and differential nonresponse and adjusted for age, sex, ethnicity, socioeconomic status, BMI, hair treatment, and time elapsed between data collection points (Wave 5 and Wave 6).

Analyses stratifying by degree of obesity revealed a stronger association between perceived weight discrimination and hair cortisol in individuals with more severe obesity (Figure 1). In the group with moderate (class I) obesity, mean hair cortisol was 17% higher in individuals who had experienced weight discrimination than those who had not, but this difference was not statistically significant (*F* = 1.40, *P* = 0.237). By contrast, among those with severe (class II/III) obesity, experiencing weight discrimination was associated with a 44% higher mean hair cortisol level; a statistically significant difference (*F* = 11.58, *P* = 0.001).

Sensitivity analyses using continuous data on perceived weight discrimination produced consistent results, with more frequent experiences of weight discrimination significantly associated with higher mean hair cortisol overall (*F* = 5.13, *P* = 0.001), and a moderating effect of obesity class showing a stronger association in participants with severe obesity (*F* = 4.92, *P* = 0.001) than in those with moderate obesity (*F* = 2.33, *P* = 0.057).

We observed no significant association between hair cortisol concentration and an aggregate variable measuring all forms of perceived discrimination (i.e., discrimination attributed to any reason rather than to weight in particular) (*F* = 1.74, *P* = 0.188).

## Discussion

This study examined the relationship between weight discrimination and hair cortisol concentration, a biomarker for chronic stress exposure. Using assays of cortisol from scalp hair, we found that people with obesity who believed they had been treated unfairly because of their weight had significantly greater exposure to cortisol over a period of approximately 2 months than those who had not experienced weight‐related discrimination. This finding is consistent with evidence from experimental studies showing increased salivary cortisol reactivity in individuals exposed to weight stigma 27, 28 and indicates that experiences of weight stigmatization and discrimination not only lead to temporary increases in cortisol, but are also associated with chronically elevated cortisol levels.

When we stratified our analysis by obesity class, we found that the association between weight discrimination and chronic stress was much more pronounced in individuals living with severe obesity than in those with moderate obesity. This may be because these people experience more extreme cases of discrimination or because they are more likely to internalize weight bias 37. Internalization of weight bias is a measure of belief in social stereotypes relating to obesity and negative self‐evaluations due to one's weight and has been shown to correlate positively with self‐reported stress 38. A previous study examining cortisol reactivity in response to manipulated weight stigma found evidence of moderation by weight perceptions, which are also associated with internalization of weight bias 37, such that a sustained elevation in cortisol levels following exposure to stigma was only seen in participants who perceived themselves as heavy 28.

This study had several strengths, including objective measures of height and weight and an indicator of chronic exposure to cortisol. There were also limitations. Discrimination was not assessed at the same time as hair sample collection, so data on weight discrimination are from 2 years earlier than the other measures in these analyses. However, previous work in this cohort has shown very high stability in BMI over time 39, so it is unlikely that this greatly influenced the present results. Although we applied weights to correct for sampling bias and nonresponse, which approximated the age and sex distribution of England; the sample was not necessarily representative based on other characteristics, such as socioeconomic position. In addition, participants were from an older population, in which levels of cortisol and experiences of weight discrimination may differ relative to younger populations 1, 40, and the sample was almost exclusively of white ethnic origin, so findings cannot be assumed to generalize. Our analyses did not take into account use of medications that might influence cortisol concentration, such as steroids or hormone replacement therapies. The cross‐sectional design meant it was not possible to determine causality, and it is therefore possible that greater cortisol exposure increases likelihood of perceiving weight discrimination as opposed to discrimination driving increases in cortisol. The incidence of discrimination is difficult to measure objectively because it relies on interpretation of the intentions of others; thus, it is possible for discrimination to occur without being perceived by the individual who is discriminated against and for discrimination to be perceived in cases where it did not occur. A person's psychological state, which may be influenced by levels of stress, may influence the way they interpret others' behavior and hence whether discrimination is perceived. However, experimental manipulations of weight stigma showing increases in cortisol reactivity following exposure to stigmatizing messages and imagery 27, 28 have indicated that discriminatory experiences are likely to play a causal role in chronically activating the HPA axis. Moreover, while the lack of an objective assessment of discrimination is an important consideration, it is possible that perceptions of stigma may themselves be salient stressors, even in the absence of “objective” discrimination.

These results provide valuable insight into the relationship between weight stigma and physiological stress. Future research could extend these findings by using longitudinal designs to explore the impact of weight discrimination on changes in cortisol concentration across multiple time points. It would also be interesting to investigate whether weight‐based discrimination has a unique effect on cortisol, as compared with other forms of discrimination. Our analysis of data on perceived discrimination in general did not show a significant association with hair cortisol concentration, which indicates that there may be something about perceived weight‐related discrimination that is particularly stressful. Weight stigma has been described as one of the “last socially acceptable forms of prejudice,” and as such, it is possible that weight‐related discrimination may be more likely to be internalized than other forms of discrimination such as racism or sexism, which are generally less socially accepted. Greater internalization of stigma has previously been shown to be associated with higher levels of stress 38.

In summary, our results support an association between weight discrimination and chronic stress, particularly in individuals living with more severe obesity. Exposure to chronically elevated cortisol levels has substantial implications for health and well‐being, and as such, experiences of weight‐based discrimination might play an important role in driving many of the adverse psychological and physiological consequences of obesity. Additionally, effects of cortisol on appetite and fat deposition make it likely that activation of the HPA axis in response to weight discrimination perpetuates a cycle of weight gain and subsequent discrimination. These findings underscore the need for initiatives to tackle the issues of weight stigma and discrimination in society in order to reduce the global burden on individuals with obesity.
